# Low health literacy limits behavioral changes during phase I cardiac rehabilitation: a multicenter clinical study

**DOI:** 10.1007/s00380-025-02589-5

**Published:** 2025-07-29

**Authors:** Yuji Kanejima, Kazuhiro P. Izawa, Masahiro Kitamura, Kodai Ishihara, Asami Ogura, Ikko Kubo, Hitomi Nagashima, Hideto Tawa, Daisuke Matsumoto, Ikki Shimizu

**Affiliations:** 1 Department of Public Health, Graduate School of Health Sciences, Kobe University, Kobe University, 10-2 Tomogaoka 7-Chome, Suma-ku, Kobe, 654-0142 Japan; 2 Cardiovascular Stroke Renal Project (CRP), Kobe, Japan; 3 Department of Rehabilitation, Kobe City Medical Center General Hospital, Kobe, Japan; 4 School of Physical Therapy, Faculty of Rehabilitation, Reiwa Health Sciences University, Fukuoka, Japan; 5 Department of Physical Therapy, Konan Women’s University, Kobe, Japan; 6 Department of Rehabilitation, Sanda City Hospital, Sanda, Japan; 7 Department of Rehabilitation, Yodogawa Christian Hospital, Osaka, Japan; 8 Department of Rehabilitation, Shinyukuhashi Hospital, Yukuhashi, Japan; 9 Department of Cardiology, Sanda City Hospital, Sanda, Japan; 10 Department of Cardiovascular Medicine, Yodogawa Christian Hospital, Osaka, Japan; 11 Department of Diabetes, Sakakibara Heart Institute of Okayama, Okayama, Japan

**Keywords:** Cardiac rehabilitation, Health literacy, Behavioral changes, Transtheoretical model, Cohort study

## Abstract

Cardiovascular diseases are strongly associated with poor lifestyle habits. Adopting healthier lifestyle habits is essential for reducing the risks associated with cardiovascular diseases. However, low health literacy (HL) may compromise assessing, using, and understanding health information, making modification of health behavior less likely. This study aimed to clarify the relationship between HL and changes in health behavior in inpatients undergoing cardiac rehabilitation. This was a multicenter cohort study of patients undergoing phase I cardiac rehabilitation. We used the 14-item Health Literacy Scale (HLS-14) to assess HL and the transtheoretical model (TTM) to assess changes in health behavior. Behavioral changes during hospitalization were defined using the stages of the TTM. A total of 428 patients who underwent cardiac rehabilitation (mean age: 73.0 years; males: 71.5%) were included. Evaluation of the behavioral changes exhibited by the participants revealed that the pre-contemplation stage (83.2%) was common at the beginning of rehabilitation, whereas contemplation (50.7%) and preparation (38.1%) stages were common at discharge. Of the 428 participants, 83.6% exhibited behavioral changes during hospitalization. Multivariate analysis revealed that HLS-14 score was a significant explanatory variable associated with changes in health behavior (odds ratio: 1.04; 95% confidence interval: 1.00–1.07). The spline curves modeling the relationship between HLS-14 score and changes in health behavior showed a positive correlation in the low HL group. HL is significantly correlated with changes in health behavior, especially in individuals with a low HL level.

**Graphical abstract:**

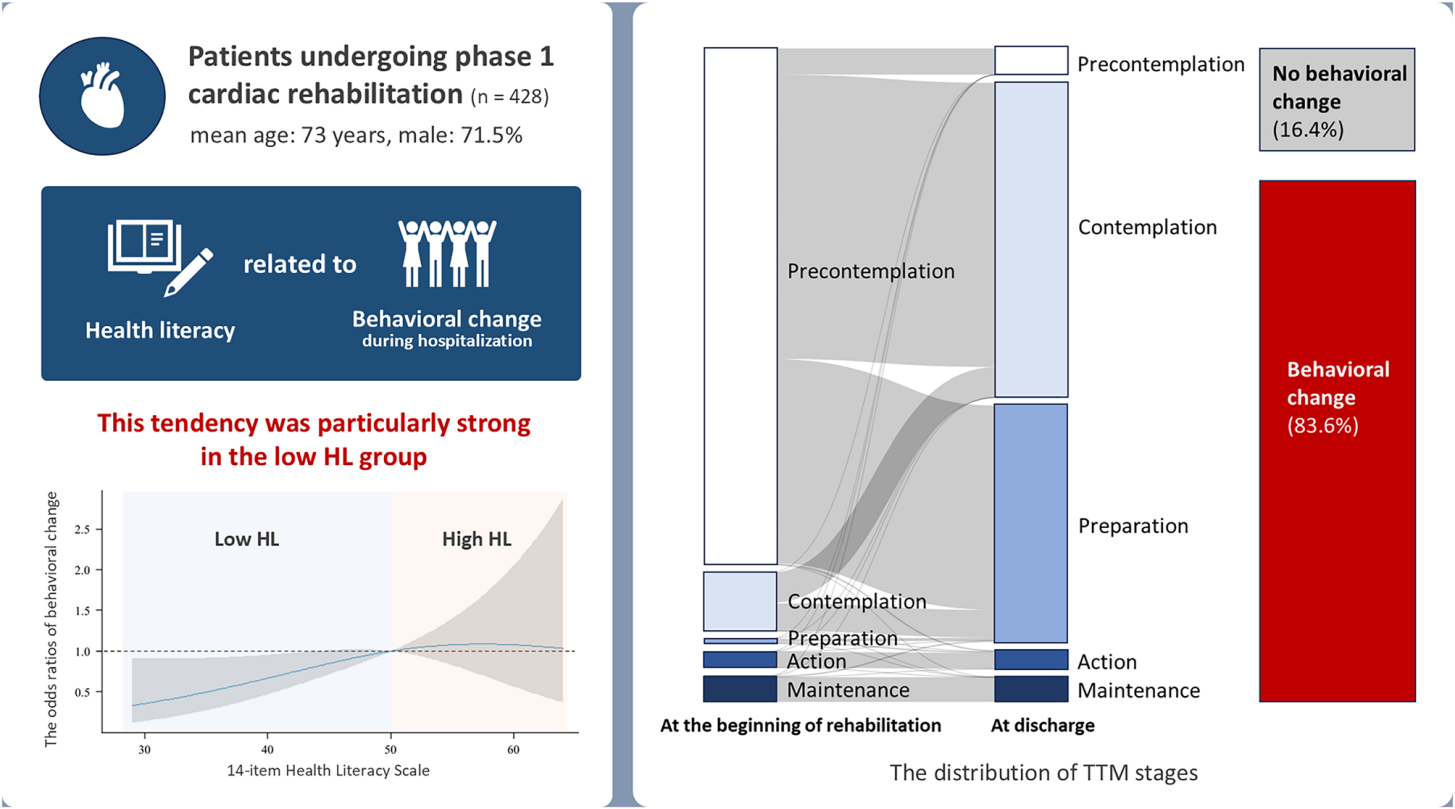

**Supplementary Information:**

The online version contains supplementary material available at 10.1007/s00380-025-02589-5.

## Introduction

Cardiovascular disease (CVD) is strongly associated with poor lifestyle habits such as physical inactivity, smoking, and unhealthy eating habits [1]. Avoiding these habits and adopting healthier behaviors can significantly reduce CVD morbidity and mortality, improve quality of life, and reduce healthcare costs [1–3]. Moreover, changes in health behavior are as important as medications in reducing the progression of heart failure [4, 5]. Cardiac rehabilitation was developed to promote positive changes in health behavior and lifestyle habits through patient education, thereby improving prognosis and ensuring comfortable and active lives [6]. However, changes in health behavior vary according to patient characteristics such as sex, race, geographical region, and economic status [1, 2]. In addition, sustaining behavioral changes long term may be challenging [7]. Therefore, it is important to personalize patient health education based on individual patient characteristics.

Health literacy (HL) refers to the cognitive and social skills that determine the motivation and ability of individuals to gain access to, understand, and use information to promote and maintain their health [8]. Low HL is associated with increased CVD risk, mortality, and readmission rates and decreased physical function and activities of daily living in patients undergoing cardiac rehabilitation [9–12]. Low HL includes reduced ability to research and understand health information, difficulty communicating health-related issues and desires, barriers to participation in health care, and lack of engagement with health care professionals [9]. Cardiac rehabilitation guidelines highlight the importance of improving individual HL and recommend that patient HL be considered when providing patient education [6, 13].

Low HL may limit changes in health behavior owing to limited access to, understanding, and use of health-related information. However, no study comprehensively investigated the relationship between HL and changes in health behavior during cardiac rehabilitation. We hypothesized that patients with low HL who are undergoing phase I cardiac rehabilitation would be less likely to initiate changes in health behavior during hospitalization than those with high HL. Therefore, this study aimed to analyze the relationship between HL and changes in health behavior in inpatients undergoing cardiac rehabilitation.

## Materials and methods

### Study design and eligibility criteria

This was a prospective cohort study conducted as part of the Kobe-Cardiac Rehabilitation Project for People around the World (K-CREW). K-CREW is a multicenter clinical study that involves four participating hospitals. The hospitals are small-to-medium-scale facilities (200–580 beds) that provide cardiac rehabilitation services. We included patients who were undergoing cardiac rehabilitation in the hospitals between October 1, 2020 and September 30, 2023. The other inclusion criterion was hospitalization for more than 5 days, not including short-term hospitalization for a medical check-up (e.g., admission for coronary angiography, percutaneous coronary intervention, ablation, or pacemaker battery replacement). The exclusion criteria were probable dementia (diagnosis or Mini-Mental State Examination score < 24), inability to walk independently, refusal to provide informed consent, hospital death, and missing or insufficient data. The primary diagnoses were divided into heart failure, ischemic heart disease, and other diseases.

### Phase I cardiac rehabilitation

Phase I cardiac rehabilitation typically includes exercise regimens and educational sessions designed for hospitalized patients [14]. The hospitals that participated in this study developed and implemented cardiac rehabilitation programs based on standard guidelines [6]. According to the guidelines, cardiac rehabilitation should be initiated within 3 days after hospitalization or cardiac surgery [6]. The exercise regimens included aerobic exercise and resistance training performed for 20–40 min daily, 5–7 days per week. The time allocations for the exercise sessions varied slightly across the hospitals, with the aerobic exercises performed for 10 to 25 min and resistance training for 10 to 20 min. Exercise intensity was adjusted using the Borg Rating of Perceived Exertion scale 11–13 or based on each patient’s anaerobic metabolic threshold [15]. The educational sessions included lectures on disease management and lifestyle modifications. Physicians, nurses, registered dietitians, pharmacists, physical/occupational therapists, and health and exercise instructors delivered lectures based on their specialties.

### Assessment of health literacy

We used the Health Literacy Scale (HLS-14) to assess the participants’ HL at the time of discharge [16]. The HLS-14 is a Japanese HL scale that consists of 14 questions divided into the following three domains: functional, communicative, and critical HL. Each question is scored on a scale of 1–5 points, with the total scores ranging from 14 to 70 points, and higher scores indicating better HL. The reliability and validity of the HLS-14 have been demonstrated previously [16]. We divided the participants into the low and high HL groups using a cutoff of 50 points [16].

### Assessment for changes in health behavior

We used the transtheoretical model (TTM) to assess participants’ changes in health behavior. TTM is a comprehensive and integrated behavioral change model based on the principle that behavioral change is a process rather than a coincidence and that different people exhibit different stages of change and readiness [17, 18]. TTM is generally used to interpret behavioral change in five stages: Pre-contemplation, which refers to a lack of intention to take action in the foreseeable future, usually measured as the next 6 months; Contemplation, which means the intention to change within the next 6 months; Preparation, which is the intention to take action in the immediate future, usually measured as the next month; Action, which refers to making specific overt lifestyle modifications within the past 6 months; and Maintenance, which means working to prevent relapse between 6 months and 5 years after initiation of lifestyle modifications [17, 19, 20]. We used the TTM stages to assess the participant lifestyle modifications, including exercise, at the beginning of rehabilitation and at discharge to determine whether the patients relapsed into poor lifestyle habits. Behavioral change was defined as advancing by one or more TTM stages at discharge compared with the beginning of rehabilitation.

### Other variables

We extracted data on the following basic characteristics from the participant medical records: age, sex, body mass index (BMI), smoking status, employment status, marital status, living situation (alone or with someone), left ventricle ejection fraction, serum hemoglobin level, creatinine level, Geriatric Nutritional Risk Index (GNRI) [21], Charlson Comorbidity Index [22], and presence of comorbidities. Several members of the cardiac rehabilitation team assessed the participants’ handgrip strength, functional independence measures (FIM), and cognitive function at discharge. In addition, one researcher collated data on medication use from the medical records system of each participating hospital.

### Statistical analysis

We conducted a comparative analysis of the low and high HL groups. First, we checked the normality of the variables using the Shapiro–Wilk test. Based on the results, we used the Student *t* test or Wilcoxon rank sum test to analyze the continuous variables, and Pearson’s Chi-squared test or Fisher’s exact test for the categorical variables. Thereafter, we used a generalized linear mixed model to analyze changes in health behavior (improved TTM stage). The following independent variables were selected based on data from previous studies (Model A): age, sex, BMI, smoking, employment status, marital status, living situation, duration of admission, primary diagnosis (heart failure or not), comorbidities (congestive heart failure, diabetes mellitus, stroke, renal dysfunction, and mild cognitive impairment [MCI]), handgrip strength, FIM, and HLS-14 score [10–12, 23, 24]. Institution ID was selected as the random effect. In addition, we checked the impact of the HLS-14 domains on behavioral changes by replacing the HLS-14 score in Model A with functional, communicative, or critical HL score. Further, we created a spline curve to model the relationship between the odds ratios (ORs) of behavioral change and HLS-14 score in Model A based on the 50-point cutoff score. Finally, we used a generalized linear mixed model (Model A) to perform a subgroup analysis of behavioral changes according to primary diagnosis.

We reported the nonparametric variables as median (interquartile range) and the parametric continuous variables as mean ± standard deviation. All analyses were conducted without any missing data. The significance level was set at p < 0.05. All statistical analyses were performed using R version 4.3.2 (The R Foundation for Statistical Computing, Vienna, Austria) for statistical analysis.

### Ethical considerations

This study was conducted in accordance with the principles outlined in the Declaration of Helsinki [25]. Ethical approval for this study was granted by Kobe University on August 12th, 2020 (No. 951–1). In addition, the ethical committee of each participating hospital approved this study. At each affiliated facility, we obtained informed consent from the patients before participation in this study, and only those who gave their consent were included.

## Results

### Basic characteristics of the participants

A total of 11,504 participants with CVD admitted to the participating hospitals during the study period were considered for inclusion into this study. Of these, 3,601 participants who did not have short-term admissions for procedures such as coronary angiography or ablation were enrolled into this study. Of these, patients with probable dementia (n = 395), those who could not walk independently (n = 491), who did not provide informed consent (n = 533), who died in the hospital (n = 139), with missing/insufficient data (n = 339), and who were ineligible for other reasons (n = 1,276) were excluded. Finally, 428 participants were included in the final analysis (Supplementary Fig. 1).

**Fig. 1:**
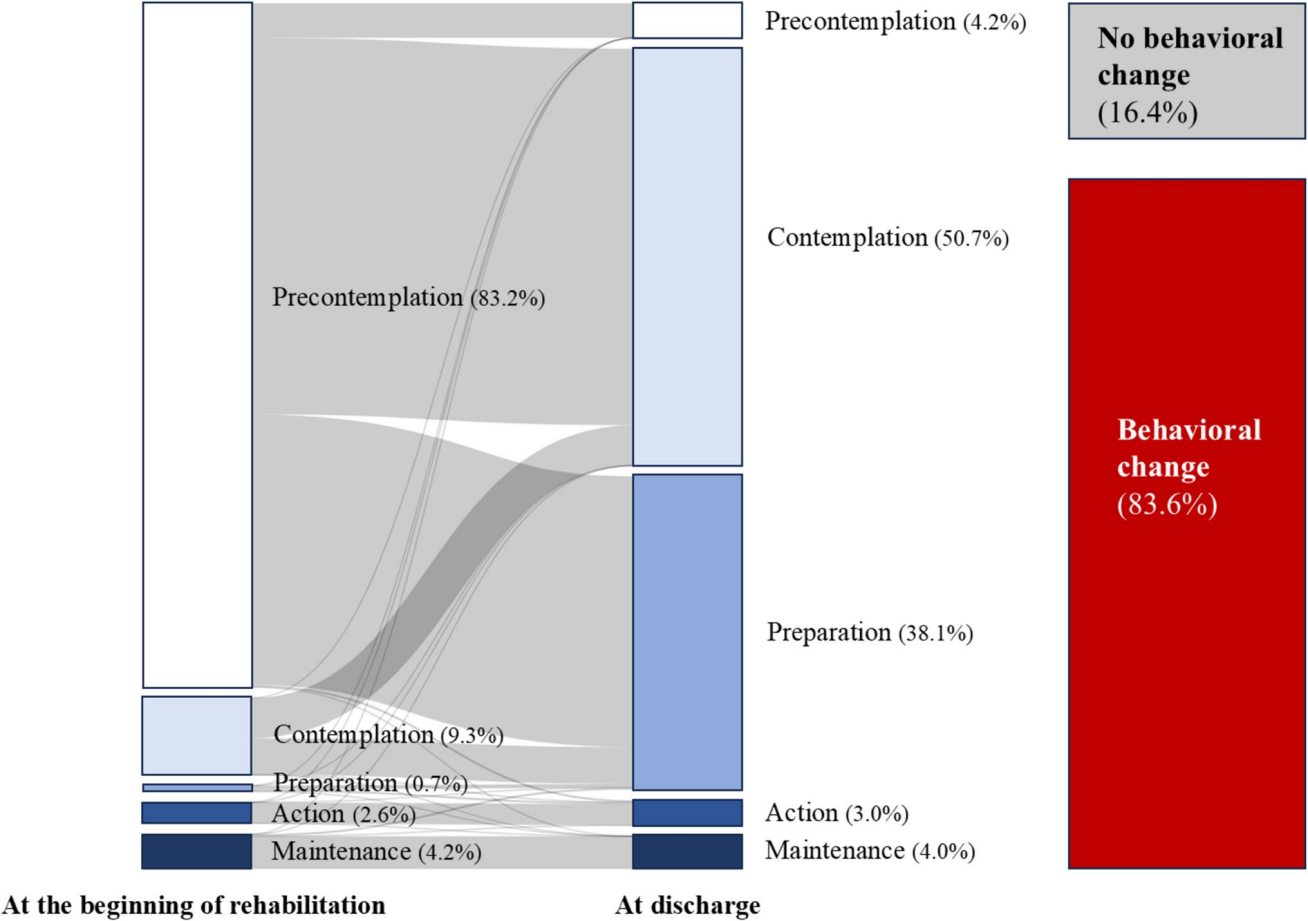
The distribution of TTM stages at the beginning of rehabilitation and discharge. The left and middle columns show the TTM stage of the overall participants at the beginning of rehabilitation and discharge. The right column shows the percentage of behavior change during hospitalization

The mean age of the participants was 73.0 [64.0, 81.0] years, and 71.5% of them were male. Regarding the primary diagnoses, 41.8% of the patients had heart failure and 57.7% had ischemic heart disease. The mean duration of admission was 15.0 [12.0, 19.0] days. The mean HLS-14 score was 47.0 [41.0, 54.0] points, and the greater proportion of the participants (59.6%) had low HL. Regarding the distribution of TTM stages, the Pre-contemplation stage was most common (83.2%) at the beginning of rehabilitation, whereas the Contemplation stage was most common (50.7%) at discharge, followed by the Preparation stage (38.1%). Of all participants, 83.6% exhibited behavioral changes during hospitalization (Fig. 1).

### Comparative analysis of the low and high health literacy groups

The participants in the low HL group were older, had a lower proportion of males, lower BMI, and a higher rate of heart failure than those in the high HL group (Table 1). In addition, the low HL group had significantly lower employment and marriage rates, higher stroke and MCI comorbidity rates, lower hemoglobin levels and GNRI, lower ACE inhibitor prescription rates, and lower grip strength and FIM scores. Furthermore, the low HL scored significantly lower than the high HL group in all domains of the HLS-14. There was no significant difference in the distribution of TTM stages at the beginning of rehabilitation; however, the high HL group exhibited a significantly more advanced overall TTM stage at discharge than the low HL group. The Contemplation stage was the most common TTM stage in the low HL group (55.3%), whereas the Preparation stage was most common in the high HL group (47.4%). Behavioral changes were less likely to occur in the low HL group than in the high HL group; however, this finding was not significant (low vs. high HL group: 81.2% vs. 87.3%, p = 0.123).

**Table 1:** Clinical characteristics of the participants in the low and high health literacy groups

	Low HL (*n* = 255)	High HL (*n* = 173)	t, Z, χ^2^ value	*p*-value ^3^
Age (years)^1^	75.0 [67.0, 84.0]	69.0 [58.0, 76.0]	5.91	< 0.01
Male ratio (%)	68.6	75.7	2.55	0.14
BMI (kg/m^2^)^1^	22.7 [19.8, 25.3]	23.7 [21.1, 25.8]	2.75	< 0.01
Employment (%)	48.2	60.7	6.43	0.02
Living with someone (%)	58.4	70.5	4.26	0.05
Smoking (%)	57.3	63.6	1.72	0.23
Marriage (%)	76.9	85.0	6.49	0.02
Duration of admission (days)^1^	15.0 [12.0, 19.0]	15.0 [11.0, 19.0]	0.85	0.40
Discharge (%)	94.9	98.3	3.57	0.12
Primary diagnosis (%)			–	0.33
Heart failure	44.7	37.6		
Ischemic heart disease	54.9	61.8		
Other diseases	0.4	0.6		
*Comorbidities (%)*				
Hypertension	68.6	61.8	2.11	0.18
Diabetes mellitus	36.5	32.9	0.56	0.52
Dyslipidemia	56.5	56.6	0.001	0.97
Congestive heart failure	38.4	33.5	1.07	0.35
Stroke	12.5	5.8	5.34	0.03
Renal dysfunction	13.3	9.2	1.67	0.26
Mild cognitive impairment	32.2	13.3	19.8	< 0.01
Charlson Comorbidity Index^1^	2.00 [1.00, 3.00]	2.00 [1.00, 3.00]	1.29	0.20
LVEF (%)^1^	53.9 [41.0, 61.0]	51.2 [39.6, 61.5]	0.71	0.48
Hemoglobin (g/dL)^1^	12.6 [11.3, 14.2]	13.6 [11.9, 14.8]	3.69	< 0.01
Creatinine (mg/dL)^1^	0.98 [0.79, 1.31]	0.94 [0.81, 1.17]	0.97	0.33
GNRI^2^	98.2 ± 11.3	101.5 ± 11.8	2.88	< 0.01
*Medications (%)*				
Beta blocker	60.4	65.9	1.33	0.29
ACE-I	21.6	32.9	6.91	< 0.01
ARB	27.5	27.2	0.004	0.95
Diuretic	60.0	52.6	2.30	0.16
Statin	69.0	69.9	0.04	0.92
Handgrip strength (kgf)^1^	24.9 [16.7, 33.0]	32.1 [22.8, 38.1]	5.60	< 0.01
Gate speed (m/s)^2^	0.88 ± 0.30	1.00 ± 0.24	4.14	< 0.01
FIM^1^	123 [109, 126]	126 [123, 126]	6.20	< 0.01
*HLS-14*				
Functional HL^1^	19.0 [15.0, 22.5]	24.0 [21.0, 25.0]	13.0	< 0.01
Communicative HL^1^	13.0 [10.0, 16.0]	19.0 [17.0, 21.0]	18.1	< 0.01
Critical HL^1^	10.0 [7.0, 12.0]	14.0 [12.0, 15.0]	14.9	< 0.01
Total score ^1^	42.0 [38.0, 46.0]	55.0 [52.0, 58.0]	21.7	< 0.01
*TTM stage at admission*			-	0.17
Precontemplation	82.4	84.4		
Contemplation	9.8	8.6		
Preparation	0.8	0.6		
Action	3.9	0.6		
Maintenance	3.1	5.8		
*TTM stage at discharge*			–	< 0.01
Precontemplation	5.9	1.7		
Contemplation	55.3	44.0		
Preparation	31.8	47.4		
Action	3.9	1.7		
Maintenance	3.1	5.2		
Behavioral change	81.2	87.3	2.38	0.12

^1^Median (Interquartile range)

^2^Mean ± Standard deviation

^3^Wilcoxon rank sum test; Student’s *t*-test; Pearson's chi-square test; Fisher's exact test

*ACE-I* angiotensin-converting enzyme inhibitor, *ARB* angiotensin II receptor blocker, *BMI* body mass index, *FIM* functional independence measure, *GNRI* geriatric nutritional risk index, *HL* health literacy, *HLS-14* 14-item Health Literacy scale, *LVEF* left ventricular ejection fraction, *TTM* transtheoretical model

### Multivariable analysis of changes in health behavior

**Fig. 2:**
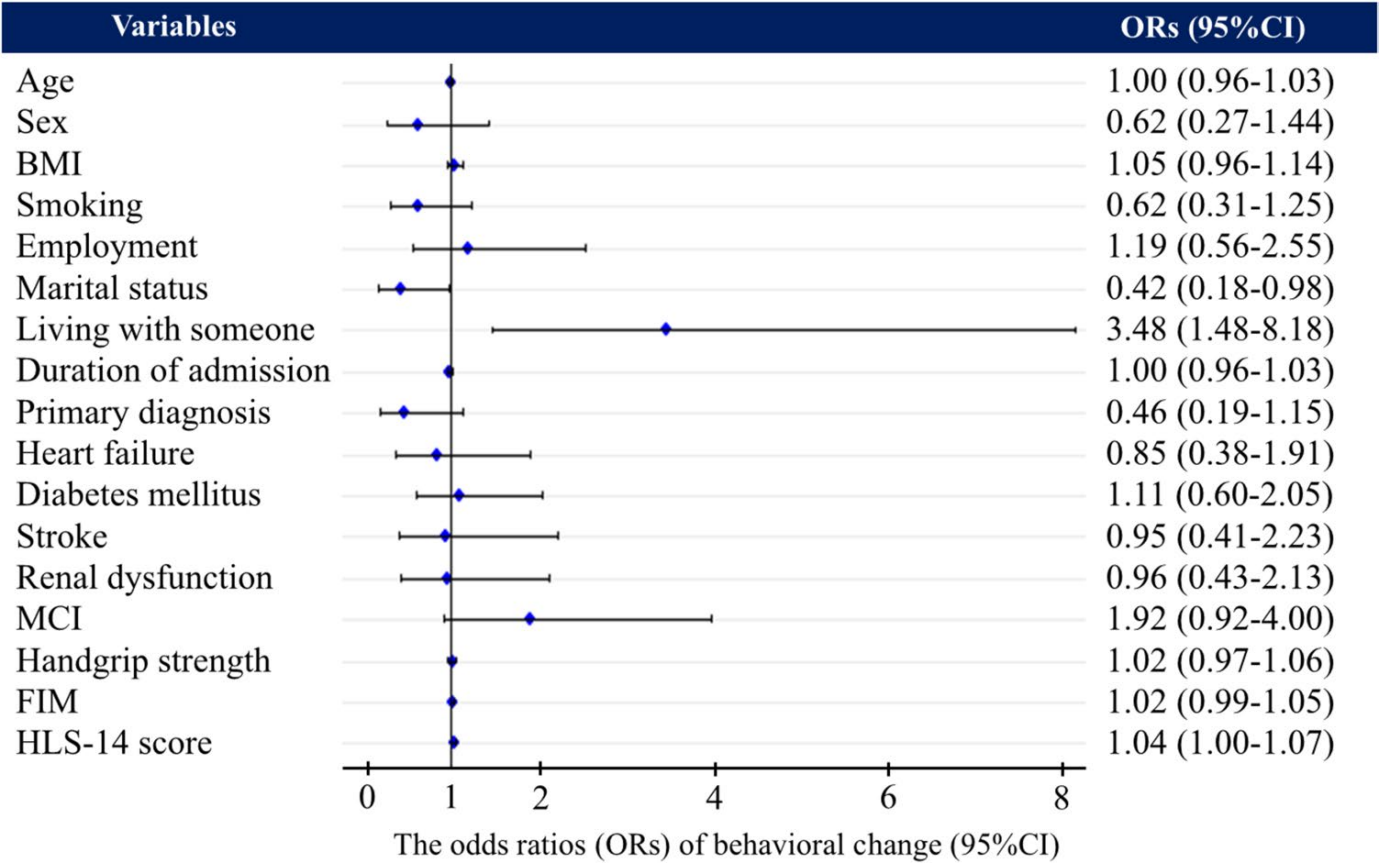
Forest plot of the generalized linear mixed model for behavior change. The results of the generalized linear mixed model for behavior change are presented in numerical and forest plots. The random effect was Institution ID. *BMI* body mass index, *CI* confidence interval, *FIM* functional independence measure, *HLS-14* 14-item health literacy scale, *MCI* mild cognitive impairment

**Fig. 3:**
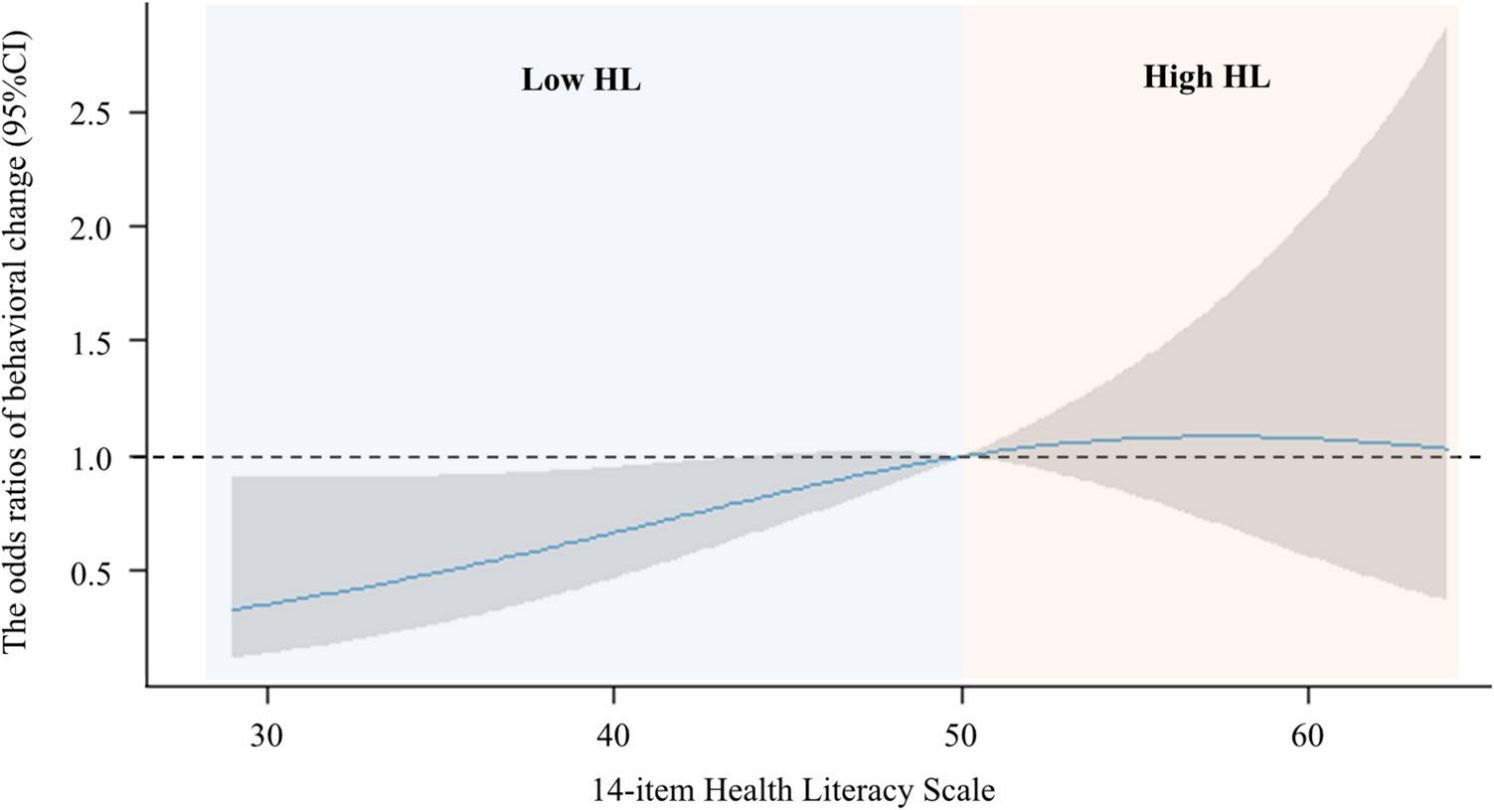
Spline curve modeling the relationship between behavior change and HLS-14. This spline curve models the relationship between behavior change and HLS-14. Explanatory variables include age, sex, BMI, smoking, employment, marriage, living together, admission duration, primary diagnosis (heart failure or not), comorbidities (congestive heart failure, diabetes mellitus, stroke, renal dysfunction, and MCI), handgrip strength, and FIM. HLS-14 scores of 50 or less indicate low HL and above 50 indicate high HL. Gray areas indicate 95% confidence intervals. *BMI* body mass index, *CI* confidence interval, *FIM* functional independence measure, *HL* health literacy, *HLS-14* 14-item health literacy scale, *MCI* mild cognitive impairment

The generalized linear mixed model revealed that HLS-14 score was a significant explanatory variable associated with changes in health behavior (OR: 1.04; 95% confidence interval [CI]: 1.00–1.07) (Fig. 2). Other variables that were significantly associated with changes in health behavioral were marital status and living with someone. The adjusted coefficient of determination for this model was 0.238. When total HLS-14 score was replaced with the domain scores in Model A, only functional HL was significantly associated with changes in health behavior (OR: 1.08; 95% CI: 1.01–1.15)) (Supplementary Table 1). The spline curves modeling the relationship between changes in health behavior and HLS-14 score showed a positive correlation in the low HL group, which had an average HLS-14 score lower than 50 points (Fig. 3). However, no positive correlation was observed in the high HL group. The subgroup analysis conducted according to primary disease revealed that HLS-14 score was significantly associated with changes in health behavior in patients with heart failure (OR: 1.06; 95% CI:1.01–1.11) but not in patients with ischemic heart disease (OR: 1.01; 95% CI: 0.95–1.07) (Supplementary Table 2).

## Discussion

The present study is the first cohort study conducted to analyze the association between HL and changes in health behavior in patients undergoing phase I cardiac rehabilitation. As we hypothesized, the results indicated that lower HL was associated with a lower likelihood of exhibiting changes in health behavior. Notably, 83.6% of the participants exhibited behavioral changes during hospitalization. At discharge, the low HL group had a higher percentage of patients in the Contemplative stage, whereas the high HL group had a greater proportion of patients in the Preparation stage. Multivariate analysis showed that HLS-14 score was significantly associated with changes in health behavior. In addition, the spline curve indicated a positive correlation between lower HLS-14 scores and lower odds ratios for changes in health behavior in the low HL group.

We compared this study with a Chinese randomized controlled trial (RCT) that was focused on behavioral changes in patients with coronary artery disease [26]. The mean age of the participants in this RCT was younger (63.3 years) than that of the participants in the present study. In addition, the proportion of males in the RCT was lower (approximately 60%) than that in the present study. Moreover, most of the patients in the intervention group in the RCT (62.5%) were in the Pre-contemplation stage at baseline. however, the Contemplation stage (39.6%) and the Preparation stage (25.0%) were more common at discharge [26]. Although these trends are similar to those observed in the present study, the percentage of the participants in the present study who advanced from the Pre-contemplation stage is higher than that in the Chinese RCT. Moreover, as hospitalization for CVD can promote changes in health behavior [27], the inpatient cardiac rehabilitation and patient education that the participants of the present study received may have facilitated improvements in their TTM stages.

In a previous study conducted to evaluate behavioral changes toward smoking during the life stages in patients with CVD, 72.0% of the patients were in the Preparation stage during hospitalization [28]. In addition, only 30% of the patients successfully avoided smoking during their living phase at 6 months. In the present study, most of the participants were in the Contemplation and Preparation stages at discharge; however, they had not yet changed their lifestyle habits. This indicates that it is important to ensure that behavioral changes are initiated and maintained in patients’ daily lives. In addition, transitioning to outpatient cardiac rehabilitation and continuing to follow-up the patient may be necessary for better behavioral modification outcomes.

Most participants with low HL in the present study were in the Contemplation stage at discharge. Provision of pertinent health information is essential for individuals in the Contemplation stage [29] as they may need access to it progress to the Preparation stage. Therefore, low HL may be an obstacle to promoting behavioral change because it may make it difficult for patients to move to the next stage if the information is provided but not understood. Approximately 80% of the patients in the present study showed improvement in TTM stage during hospitalization. However, it should be noted that TTM stage is reversible and patients may return to the previous stages [19]. Interventions targeting behavioral change often result in a noticeable initial change but do not generally lead to long-term maintenance of the behavioral changes [1]. People with low HL typically have limited access to health information and services [9, 10]. In addition, follow-up after discharge is difficult, and participants with low HL are anticipated to have difficulty improving or maintaining behavioral changes after hospitalization.

The TTM is based on a spectrum concept of the process of change, and it is recommended that this process be appropriate for the stage of behavioral change [19]. In the present study, most patients transitioned from the Pre-contemplation stage to the Contemplation and Preparation stages. At these stages, the cognitive processes of change are important for modifications of behavioral habits. The cognitive processes of change include increased consciousness, dramatic relief, environmental evaluation, self-reevaluation, and social liberation [30], which generally require basic HL. Therefore, behavioral change may be associated with functional HL only, and the results of the multivariate analysis, including the HLS-14 domains performed in the present study, supported this finding. A process of behavioral change is required for the progression to the Action or Maintenance stage. This process of change includes counterconditioning, supportive relationships, reinforcement management, self-liberation, and stimulus control [30]. Patients may require more advanced HL, such as access to and use of health information, to experience these processes. It should be noted that medical staff are likely to overestimate patients’ HL [31]. Therefore, appropriate methods and interventions should be considered after assessing HL and TTM stages to promote more significant behavioral changes.

The subgroup analysis conducted in the present study revealed an association between HL and behavioral changes in the participants with heart failure. Previous reviews have shown that higher HL is associated with greater knowledge of the disease in patients with heart failure [10, 32]. Knowledge of diseases may facilitate advancement through the TTM stages. Notably, self-efficacy is also included in the TTM spectrum concept. Self-efficacy is relevant to self-care in patients with heart failure [32]. Promoting behavioral change is likely to increase self-efficacy and enhance self-care in patients with heart failure, leading to improved outcomes such as prevention of morbidity and rehospitalization. Because some of the participating hospitals in the present study provide heart failure education to patients with heart failure, it is possible that the disease-specific education may have facilitated behavioral changes. In addition, multivariate analysis in this study showed that living with someone else tends to lead to behavioral change. A previous study stated that approaching family members is an effective way to promote behavioral change in patients [33]. Therefore, instruction that includes family members may be practical for participants with low HL.

This study has some limitations. First, many patients were excluded from the final analysis because they were hospitalized for short periods for reasons other than cardiac rehabilitation, such as coronary angiography or ablation. Second, we included participants with different types of CVD as primary diagnoses. As each disease has its distinct background and clinical characteristics, our results should be interpreted with caution. Third, we included participants with MCI in this study. However, it should be noted that we used the HLS-14 and TTM questionnaires for subjective assessments. Although MCI was included in the multivariate analysis as an explanatory variable, the reliability of these questionnaire assessments may be low. In addition, as the HLS-14 was used for the assessment of HL at discharge, the participants’ HL may have been influenced by the interventions they received during hospitalization. Furthermore, other factors relevant to HL, such as economic status in social contexts, were not considered. Finally, this study focused only on changes in health behavior during hospitalization. Most participants were discharged without reaching the Action stage in this study. Therefore, future studies will be needed to evaluate trends in HL and changes health behavior after discharge, including whether participants transition to the Action stage.

## Discussion

This study was conducted to analyze the association between changes in health behavior and HL in patients undergoing phase I cardiac rehabilitation. During hospitalization, 83.6% of the participants exhibited changes in their health behaviors. As hypothesized, multivariate analysis revealed that the HLS-14 score was significantly associated with changes in health behavior. In addition, the spline curve showed a positive correlation between HLS-14 score and behavioral changes in participants with low HL. At discharge, most participants with low HL were in the Contemplation stage, whereas those with high HL were in Preparation stage. These findings indicate that clinicians should consider interventions that encourage changes in health behavior in patients with low HL to facilitate advancement to the Preparation stage during hospitalization. In addition, promoting behavioral changes in the Action stage and beyond is important. However, further research is needed to evaluate the transition and maintenance of changes in health behavior after discharge.

## Supplementary Information

Below is the link to the electronic supplementary material.

Supplementary file1 (TIF 278 KB)

Supplementary file2 (DOCX 29 KB)

Supplementary file3 (DOCX 29 KB)

## Data Availability

The deidentified participant data cannot be shared owing to ethical and institutional restrictions.
