# Supplementary figures and images for: Low health literacy limits behavioral changes during phase I cardiac rehabilitation: a multicenter clinical study
Source: Heart Vessels. 2025 Jul 29;41(1):48–57. doi: 10.1007/s00380-025-02589-5 (PMC12795910; doi:10.1007/s00380-025-02589-5)

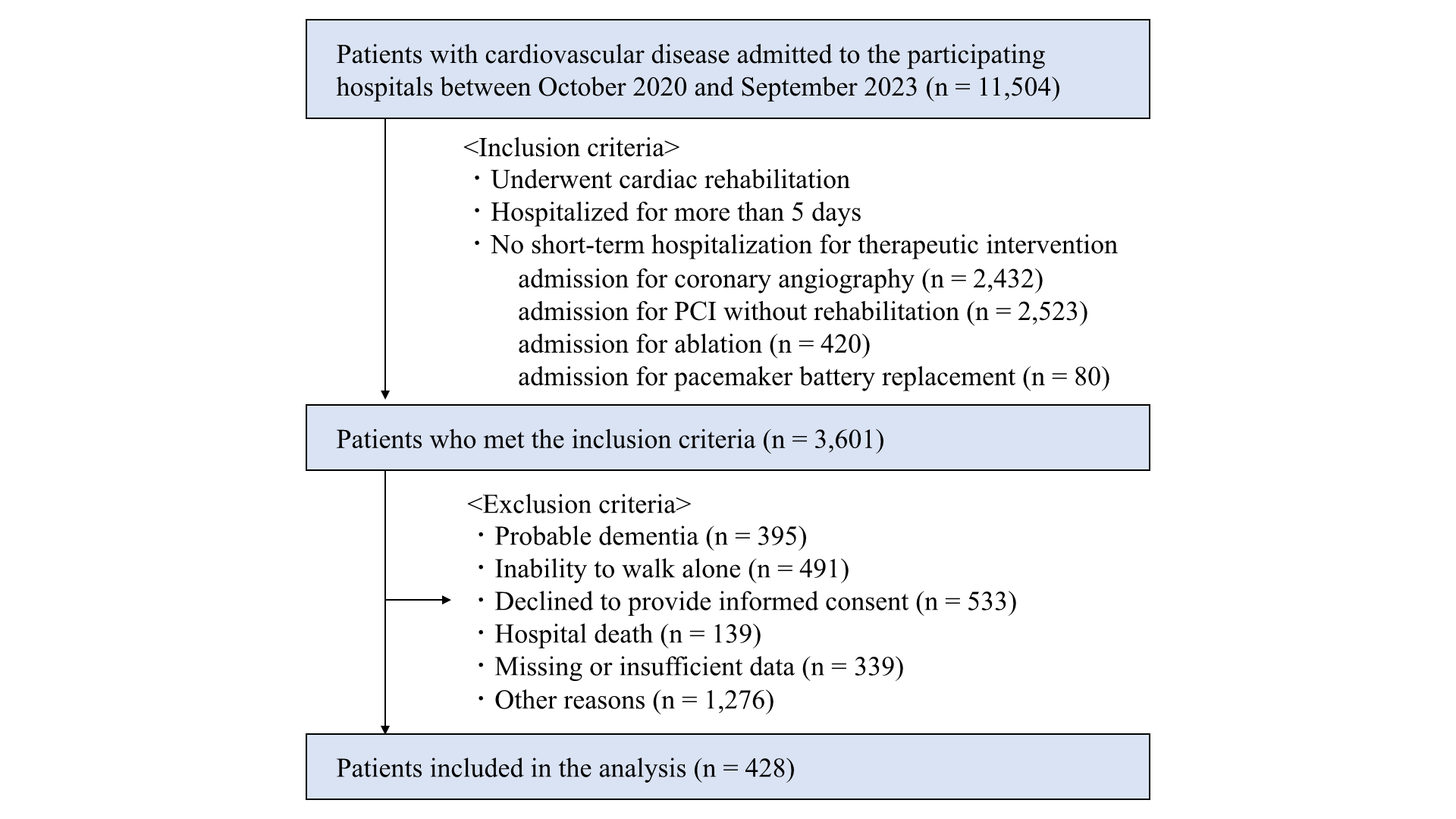

Supplement: Supplementary file 1 — Supplementary file1 (TIF 278 KB) [file 380_2025_2589_MOESM1_ESM.tif]
