# Supplementary material for: Low health literacy limits behavioral changes during phase I cardiac rehabilitation: a multicenter clinical study
Source: Heart Vessels. 2025 Jul 29;41(1):48–57. doi: 10.1007/s00380-025-02589-5 (PMC12795910; doi:10.1007/s00380-025-02589-5)
Supplement: Supplementary file 2 — Supplementary file2 (DOCX 29 KB) [file 380_2025_2589_MOESM2_ESM.docx]

**Supplementary Table 1. Generalized linear mixed models for the assessment of behavioral changes including the HLS-14 domains**

| Variables | Odds ratios | 95% CI | t value | p-value |
| --- | --- | --- | --- | --- |
| HLS-14 score was replaced with functional HL score in Model A | | | | |
| **Functional HL** | 1.08 | (1.01-1.15) | 2.33 | 0.02 |
| HLS-14 score was replaced with communicative HL score in Model A | | | | |
| **Communicative HL** | 1.01 | (0.95-1.08) | 0.38 | 0.70 |
| HLS-14 score was replaced with critical HL score in Model A | | | | |
| **Critical HL** | 1.06 | (0.98-1.16) | 1.41 | 0.16 |

^1^ Model A: age, sex, BMI, employment status, living with someone, smoking status, marital status, duration of admission, congestive heart failure, diabetes mellitus, stroke, renal disease, MCI, handgrip strength, FIM, and HLS-14 score

Abbreviations: BMI, body mass index; CI, confidence interval; FIM, functional independence measure; MCI, mild cognitive impairment; HL, health literacy; HLS-14:14-item Health Literacy Scale
