# Supplementary material for: Low health literacy limits behavioral changes during phase I cardiac rehabilitation: a multicenter clinical study
Source: Heart Vessels. 2025 Jul 29;41(1):48–57. doi: 10.1007/s00380-025-02589-5 (PMC12795910; doi:10.1007/s00380-025-02589-5)
Supplement: Supplementary file 3 — Supplementary file3 (DOCX 29 KB) [file 380_2025_2589_MOESM3_ESM.docx]

**Supplementary Table 2. The generalized linear mixed model for subgroup analysis of behavioral change according to primary diagnosis**

| Variables | Odds ratios | 95% CI | t value | p-value |
| --- | --- | --- | --- | --- |
| Subgroup: Primary disease (with model A) | | | | |
| Heart failure (n = 179) | | | | |
| **HLS-14** | 1.06 | (1.01-1.11) | 2.21 | 0.03 |
| Ischemic heart disease (n = 247) | | | | |
| **HLS-14** | 1.01 | (0.95-1.07) | 0.32 | 0.75 |

^1^ Model A: age, sex, BMI, employment status, living with someone, smoking status, marital status, admission duration, congestive heart failure, diabetes mellitus, stroke, renal disease, MCI, handgrip strength, FIM, and HLS-14 score

BMI, body mass index; CI, confidence interval; FIM, functional independence measure; MCI, mild cognitive impairment; HLS-14:14-item Health Literacy Scale
